# The Role of the Ventromedial Prefrontal Cortex in Preferential Decisions for Own- and Other-Age Faces

**DOI:** 10.3389/fpsyg.2022.822234

**Published:** 2022-03-11

**Authors:** Ayahito Ito, Kazuki Yoshida, Ryuta Aoki, Toshikatsu Fujii, Iori Kawasaki, Akiko Hayashi, Aya Ueno, Shinya Sakai, Shunji Mugikura, Shoki Takahashi, Etsuro Mori

**Affiliations:** ^1^Research Institute for Future Design, Kochi University of Technology, Kochi, Japan; ^2^Faculty of Health Sciences, Hokkaido University, Sapporo, Japan; ^3^Graduate School of Humanities, Tokyo Metropolitan University, Tokyo, Japan; ^4^Kansei Fukushi Research Institute, Tohoku Fukushi University, Sendai, Japan; ^5^Department of Behavioral Neurology and Cognitive Neuroscience, Graduate School of Medicine, Tohoku University, Sendai, Japan; ^6^Division of Image Statistics, Tohoku Medical Megabank Organization, Sendai, Japan; ^7^Department of Diagnostic Radiology, Graduate School of Medicine, Tohoku University, Sendai, Japan

**Keywords:** face, subjective value, preference, connectivity, functional magnetic brain imaging, own-age, other-age

## Abstract

Own-age bias is a well-known bias reflecting the effects of age, and its role has been demonstrated, particularly, in face recognition. However, it remains unclear whether an own-age bias exists in facial impression formation. In the present study, we used three datasets from two published and one unpublished functional magnetic resonance imaging (fMRI) study that employed the same pleasantness rating task with fMRI scanning and preferential choice task after the fMRI to investigate whether healthy young and older participants showed own-age effects in face preference. Specifically, we employed a drift-diffusion model to elaborate the existence of own-age bias in the processes of preferential choice. The behavioral results showed higher rating scores and higher drift rate for young faces than for older faces, regardless of the ages of participants. We identified a young-age effect, but not an own-age effect. Neuroimaging results from aggregation analysis of the three datasets suggest a possibility that the ventromedial prefrontal cortex (vmPFC) was associated with evidence accumulation of own-age faces; however, no clear evidence was provided. Importantly, we found no age-related decline in the responsiveness of the vmPFC to subjective pleasantness of faces, and both young and older participants showed a contribution of the vmPFC to the parametric representation of the subjective value of face and functional coupling between the vmPFC and ventral visual area, which reflects face preference. These results suggest that the preferential choice of face is less susceptible to the own-age bias across the lifespan of individuals.

## Introduction

Age has a prominent effect on face perception (Korthase and Trenholme, 1982; McLellan and Mckelvie, 1993; Perrett et al., 1998; Zebrowitz et al., 2003; Rhodes, 2006). Own-age bias is a well-known bias that reflects the effects of age on face perception (Anastasi and Rhodes, 2005; Rhodes and Anastasi, 2012). Previous studies have indicated that own-age faces are better recognized and remembered than other-age faces (Wright and Stroud, 2002; Anastasi and Rhodes, 2005). For example, Wright and Stroud (2002) showed that older culprits were better recognized by older persons than by young persons, whereas young culprits were better recognized by young persons than by older persons. Furthermore, a seminal meta-analysis conducted by Rhodes and Anastasi (2012) showed that the recognition memory for own-age faces is better than that for other-age faces across the lifespan of individuals. These results have been explained by the increased contact hypothesis (i.e., a higher frequency of contact with own-group individuals increases the expertise of face perception) (Chiroro and Valentine, 1995) and/or socio-cognitive accounts (i.e., in-group/out-group categorization of faces results in memory bias and own-group faces are better remembered than other-group faces) (Bernstein et al., 2007). Thus, own-age bias is considered to be rooted in the importance of or experience with one’s own age group in their daily lives, and the amount of exposure to a certain age group modulates the perceptual expertise of faces within the group (Ebner and Johnson, 2009; He et al., 2011; Macchi Cassia et al., 2012).

However, it remains unclear whether own-age bias exists in facial impression formation. Facial impressions are a fundamental aspect of human society, and their influence varies from mate preference to the results of political elections (Buss, 1985; Todorov et al., 2005; Todd et al., 2007; Berggren et al., 2010). Recently, researchers found that own-age bias existed in visual attention and emotion recognition, which appeared to be related to the own-age bias in face preference (Ebner et al., 2011c, 2013; He et al., 2011). For example, a previous eye-tracking study that employed a passive face viewing task revealed that people see own-age faces longer than other-age faces (He et al., 2011). Another study that used an emotion identification task also supported this finding (Ebner et al., 2011c). Although the own-age bias in visual attention is yet to be determined (Strickland-Hughes et al., 2020), own-age bias may affect facial preference via heightened visual attention toward own-age faces.

When people choose a face that they prefer from two alternatives, the subjective value of each face is computed, and these values are compared and preferential choices are made (Levy and Glimcher, 2011). Previous studies using functional magnetic resonance imaging (fMRI) have shown that the ventromedial prefrontal cortex (vmPFC) computes value signals (Chib et al., 2009; Lebreton et al., 2009; Hare et al., 2010; Ito et al., 2016; Suzuki et al., 2017; Suzuki and O’Doherty, 2020; Pessiglione and Daunizeau, 2021). Specifically, it has been argued that the brain valuation system (BVS), including the vmPFC, parametrically represents subjective values of faces and predicts later preferential choices (Lebreton et al., 2009). Other strands of evidence suggest that gaze biases preferential choice by changing attention-dependent relative value signals of choice options (Shimojo et al., 2003; Armel et al., 2008; Lim et al., 2011). It has been shown that exogenous changes in gaze duration can bias participants’ decisions (Shimojo et al., 2003; Armel et al., 2008), suggesting that the brain computes attention-dependent relative value signals. Lim et al. (2011) demonstrated that the vmPFC is associated with this value computation. Thus, different gaze patterns among people for own-age faces and other-age faces may bias subjective ratings and vmPFC activity for own-age faces. Although a previous study suggested the possibility of the existence of own-age bias in the representation of the subjective value of faces (Ito et al., 2016), this question has rarely been explicitly addressed.

To formally investigate whether the own-age bias exists in face preference, we used a drift-diffusion model (DDM), which is a type of sequential sampling model that assumes that decision making is a process comprising a noisy accumulation of evidence from a stimulus (Ratcliff and McKoon, 2008; Ratcliff et al., 2016). The DDM has been widely used to represent perceptual decision making processes, and parameters estimated from the distributions of choice probabilities and reaction times provide deeper insights into choice features among participants (Ratcliff et al., 2009, 2016; Brunton et al., 2013). In recent years, the DDM has been applied to various value-based decision making tasks, such as preferential choice of food items, and it elaborates the neurocognitive mechanisms underlying the choice processes (Milosavljevic et al., 2010; Krajbich and Rangel, 2011; Polanía et al., 2014; Lopez-Persem et al., 2016). Previous studies that employed DDMs have repeatedly reported that the vmPFC is involved in evidence accumulation during value-based choices (Basten et al., 2010; Lim et al., 2011; Hunt et al., 2012; Gherman and Philiastides, 2018). Intriguingly, the contribution of the vmPFC to face preference has also been reported in recent fMRI studies (Lebreton et al., 2009; Ito et al., 2016; Murakami et al., 2018). Thus, a DDM analysis can clarify not only the psychological processes involved in value-based choice but also whether the role of the vmPFC in evidence accumulation can be affected by the own-age bias.

We re-analyzed three datasets from two published and one unpublished fMRI study, all of which employed the same task (Ito et al., 2016; Murakami et al., 2018). The task consisted of a pleasantness-rating task during fMRI scanning and a preferential choice task after fMRI. During the preferential choice task, participants performed a two-alternative forced-choice task, which is frequently used in DDM studies (Krajbich and Rangel, 2011; Lim et al., 2011; Lopez-Persem et al., 2016). First, we applied the DDM to the behavioral data from the preferential choice task and sought to identify parameters that reflect the own-age effect. We then investigated whether the vmPFC showed patterns associated with the own-age bias. Since previous fMRI studies that used DDMs showed the contribution of the functional coupling of the vmPFC and fusiform gyrus toward value representation, we also sought to identify a functional network centered on the vmPFC, which is associated with face preference, and to investigate whether these regions also show an own-age effect.

## Materials and Methods

### Participants

The data of 116 participants from three datasets were included in the present study (study 1: 52 young males, mean age 21.8 years [range, 20–27]; study 2: 16 young females and 16 males, mean age 21.3 years [range, 20–25]; study 3: 16 older females and 16 males, mean age 68.3 years [range, 61–74]). Studies 1 and 2 have been published previously, and both studies included young participants (Ito et al., 2016; Murakami et al., 2018), whereas study 3 was an unpublished study that employed older participants. The older participants underwent mini-mental state examination, in which the most common cutoff scores are 23 and 24 (Mori et al., 1985; Tsoi et al., 2015). The results showed that they had normal cognitive function (min = 25, max = 30; mean score, 28.3 ± 2.0). Four other older participants (three older females and one older male) were excluded from the analysis because they had a cough, experienced technical problems during the experiment, or had asymptomatic infarction. No pathological findings were identified in the brains of the other participants. All participants had normal or corrected-to-normal vision. After the participants received a detailed description of the study, they provided written informed consent in accordance with the tenets of the Declaration of Helsinki. The protocol of study 1 was approved by the Ethical Committee of Hokkaido University, whereas those of studies 2 and 3 were approved by the Ethical Committee of Tohoku University.

### Stimuli and Tasks

The same stimulus set was used across the three studies, and details of stimulus preparation are shown in the original report (Ito et al., 2016). The stimulus set comprised the faces of 64 older males, 64 older females, 64 young males, and 64 young female volunteers. A separate group of 13 young volunteers who did not participate in the fMRI study rated 256 facial photographs using a 10-point scale for pleasantness. The mean pleasantness score was ranked within the four stimulus groups (i.e., older males, older females, young males, and young females). Within each stimulus group, the photographs ranked “n” (*n* = 1–32) were paired with the photographs ranked “n + 32,” which resulted in 32 pairs of photographs per group.

The experiment consisted of two tasks: a pleasantness-rating task during fMRI scanning and a preference-choice task after the fMRI scanning. For the pleasantness-rating task during the fMRI scan, each of the 256 face photographs was presented in a random order. Each stimulus was presented for 2.5 s, and the inter-stimulus interval, during which the cross-fixation was constantly presented, ranged between 3.5 and 11.5 s to maximize the efficiency of the event-related design (Dale, 1999). The pleasantness-rating task was divided into four consecutive runs, each lasting approximately 10 minutes. The participants were asked to rate each face based on how pleasant or unpleasant it was (study 1: 8-point scale; studies 2 and 3: 5-point scale). The preference-choice task was performed outside the scanner immediately after the fMRI. The 128 pairs of photographs were displayed as two side-by-side photographs, and the participants were asked to choose the face that they preferred by pressing one of two buttons. The positions of the two photographs within each pair were counterbalanced across participants.

### Neuroimaging Data Acquisition

A T2*-weighted echo planar imaging (EPI) sequence sensitive to blood oxygenation level-dependent (BOLD) contrast was used for functional imaging with the following parameters: repetition time = 2,500 ms, echo time = 30 ms, flip angle = 90°, acquisition matrix = 80 × 80, field of view = 240 mm, in-plane resolution = 3 × 3 mm, slice thickness = 3 mm, and interslice gap = 0.5 mm (number of axial slices: 42 for study 1, 43 for studies 2 and 3). An acquisition sequence tilted at 30° to the intercommissural (anterior commissure-posterior commissure) line was used to recover the magnetic susceptibility-induced signal losses due to the sinus cavities (Deichmann et al., 2003). A high-resolution (spatial resolution 1 × 1 × 1 mm) structural image was also acquired using a T1-weighted magnetization-prepared rapid-acquisition gradient echo pulse sequence. Each participant’s head motion was restricted using a firm padding that surrounded the head. EPI images were acquired over four consecutive runs. The first four scans in each run were discarded to allow for equilibration effects.

### Preprocessing

Preprocessing was performed using the Statistical Parametric Mapping 12 software (Wellcome Department of Imaging Neuroscience, London, United Kingdom). All volumes acquired from each participant were realigned to correct for small movements that occurred between scans. This process generated an aligned set of images and a mean image for each participant. The realigned images were subsequently corrected for different slice acquisition times. Each participant’s T1-weighted structural MRI was co-registered to the mean of the realigned EPI images and segmented to separate the gray matter, which was normalized to the gray matter in a template image based on the Montreal Neurological Institute (MNI) reference brain (resampled voxel size, 2 × 2 × 2 mm). Using the parameters from this normalization process, the EPI images were subsequently normalized to the MNI template and smoothed using an 8-mm full-width, half-maximum Gaussian kernel.

### Statistical Analysis of the Imaging Data

We employed four generalized linear models (GLMs) to analyze the fMRI data. All GLMs incorporated only one event per trial, which was the onset of face presentation. In GLM 1, pleasantness-rating scores were entered as a parametric regressor which revealed brain regions associated with the representation of subjective pleasantness of faces. Thus, GLM 1 included the raw value of (i.e., not mean-centered) pleasantness scores as parametric regressors. GLM 2 was used to compare brain activity between older and young faces. In GLM 2, trials were sorted based on stimulus age, with no parametric modulation. GLM 3 was applied in the psycho-physiological interaction (PPI) analysis, which identified a network centered on the vmPFC associated with the representation of the subjective value of a faces (i.e., preference). We used the coordinates of the group maximum identified in the parametric modulation analysis (study 1, *x* = –2, *y* = 36, *z* = –8; study 2, *x* = 6, *y* = 38, *z* = –8; study 3, *x* = –2, *y* = 44, *z* = –6). The coordinates served as a starting point for identifying a nearby local maximum in each participant-specific dataset. At the individual level, we identified the local activation peak within a sphere with a radius of 16 mm around the group maximum. We then extracted the first eigenvariate of the BOLD response in each participant within a sphere with a radius of 4 mm around the individual activation peak. Participants who did not demonstrate activation in the seed region at a liberal threshold of *p* < 0.05, uncorrected for multiple comparisons, were excluded from the PPI analysis (study 1, two participants; study 2, eight participants; study 3, nine participants). This GLM included the following three regressors: (1) first eigenvariate of the vmPFC; (2) a regressor specifying a psychological variable which codes preference convolved with canonical HRF; and (3) an interaction term between the two variables. The PPI analysis generated a contrast that represented the regions that exhibited stronger functional connectivity with the vmPFC for the preferred faces than for the non-preferred faces. A group-level random-effects analysis was subsequently performed by applying one-sample *t*-tests to the first-level *t*-maps. Events that involved multiple responses or no responses were modeled as events of no interest. A high-pass filter of 1/128 Hz was used to remove low-frequency noise, and an autoregressive (1) model was used to correct for temporal autocorrelations. For all whole-brain analyses, the threshold of significance was set at *p* < 0.05 (family wise error corrected for multiple comparisons at the voxel level), unless otherwise specified. The peak voxels of clusters that exhibited reliable effects were reported in the MNI coordinates. GLM 4, which employed regressors specifying the onset of each face, was used to extract the percent signal change for each stimulus face, and was subsequently applied in the analysis using the DDM as detailed in the next section.

### Drift-Diffusion Model

To examine the own-age bias in face preference, we first applied the DDM to our behavioral data. The DDM assumes that the noisy evidence for a decision accumulates over time at a certain speed (drift rate: *v*), and that the choice is executed when this accumulating evidence crosses one of two boundaries (decision threshold: *a*). We performed a hierarchical Bayesian estimation of DDM parameters for each participant using the hierarchical DDM (HDDM) 0.6.0 toolbox (Wiecki et al., 2013) implemented in Python 3^1^. If a face that had been rated as more pleasant than the paired face was chosen in the preferential choice task (i.e., consistent choice), the corresponding trial was deemed to be a correct trial. On the other hand, if a face that had been rated as less pleasant than the paired face was chosen (i.e., inconsistent choice), the corresponding trial was considered an erroneous trial. Choice trials that contained two equally rated photographs were excluded from the analysis (study 1: 9.1 ± 2.9%, study 2: 11.2 ± 3.7%, study 3: 15.2 ± 6.2%). To ensure the independence of the estimated parameter, each participant’s data were fit separately. Our model had three free parameters: non-decision time (t), decision threshold (a), and drift rate (v). The model was fit to accuracy-coded data (i.e., the upper boundary indicates a correct (consistent) choice, which is predicted by the rating data, while the lower boundary indicates an incorrect (inconsistent) choice, which is not predicted by the rating data), and the starting point was fixed at a/2. The HDDM used Markov chain Monte Carlo sampling to approximate the posterior distribution over parameter estimates. For parameter estimation, three chains were run each with 2,000 samples, and the first 500 samples in each run were discarded as burns in. In addition, Gelman and Rubin’s *-R* for each parameter was calculated to assess the convergence. This value is supposed to be close to 1 and not exceed 1.1 if convergence is successful. The mean posterior estimate parameters of each participant were extracted for subsequent statistical tests.

We also applied the HDDM using neuroimaging data to examine whether the areas related to face preference were associated with own-age bias in the preferential choice task. We used MarsBaR software^2^ to extract the activity in the regions of interest (ROIs), which was then normalized on a within-participant basis. Percent signal changes were extracted from the vmPFC and visual areas (fusiform and occipital gyri), which showed greater functional coupling with the vmPFC when participants were presented with preferred faces. To avoid the double dipping problem (Kriegeskorte et al., 2009), the percent signal change of the vmPFC was extracted from a spherical mask with a radius of 6 mm centered on the coordinates *x* = –10, *y* = 44, and *z* = –8, which were obtained from a previous study that showed that the vmPFC plays a critical role in the evaluation of faces (Lebreton et al., 2009). Percent signal changes in the visual areas were extracted from the masks of the fusiform face area (FFA) (*x* = –37, *y* = –57, *z* = –18 for the left hemisphere and *x* = 40, *y* = –48, *z* = –22 for the right hemisphere) and occipital face area (OFA) (*x* = –36, *y* = –75, *z* = –12 for the left hemisphere and *x* = 40, *y* = –73, *z* = –14 for the right hemisphere), which have been identified in a previous study (Julian et al., 2012). We applied the HDDMRegressor (Wiecki et al., 2013) to the preference choice task to examine whether the trial-by-trial brain activity in each ROI could modulate parameters that showed significant differences between older and young faces. First, we calculated the difference in percent signal change between chosen and unchosen faces by subtracting the percent signal change of the unchosen face from that of the chosen face. This calculation was performed for each face pair used in the preference choice task. Then, the difference in percent signal change between chosen and unchosen faces in each trial face pair was entered as an explanatory variable for each trial, and HDDMRegressors were estimated. In this analysis, participant IDs were included in the Bayesian hierarchical model. Therefore, the distributions of the DDM parameters, such as the intercept of the drift rate, allowed for individual differences within the group in their posterior distributions. For the regression, three chains were run each with 3,000 samples, and the first 1,000 samples in each run were discarded to improve convergence. The intercept and slope in each ROI for estimating the DDM parameters were calculated for each simulation, and the average values of 6,000 samples were reported. Convergence was assessed using Gelman and Rubin’s *-R* for each estimated parameter, including the intercept and slope. Furthermore, to formally test whether the vmPFC is differentially involved in evidence accumulation of the preferential choice for own-age faces, we performed a supplementary HDDMRegressor analysis that modeled trial-by-trial effects as follows: drift rate ∼ vmPFC activity + face age + vmPFC activity × face age. Here, both own-age and other-age face trials were included in a single model for each study (face age was dummy coded: 1 for own-age and –1 for other-age face), and the own-age bias (i.e., a greater slope for own-age faces relative to other-age faces) was operationalized as a positive regression coefficient for the interaction term. All other analytical settings were identical to those of the aforementioned HDDMRegressor analysis. In addition, we aggregated the results across studies using the *meta* package in the R environment (Schwarzer et al., 2015). We used the mean values of the estimated regression coefficients indicating the own-age bias and standard deviations of the three studies (i.e., the outputs of the HDDMRegressor analysis) to compute the pooled mean by considering the between-study variance (similar to a random-effect meta-analysis). This analysis was performed *post hoc* after observing the statistical results of single studies (i.e., the results should be viewed as a basis for future research rather than for drawing conclusions from the present study). Also, the data used in the three studies are all available as datasets collected by us with regard to testing the neural own-age bias (i.e., there was no selection bias or intentional stopping during data collection).

## Results

### Behavioral Data

**FIGURE 1:**
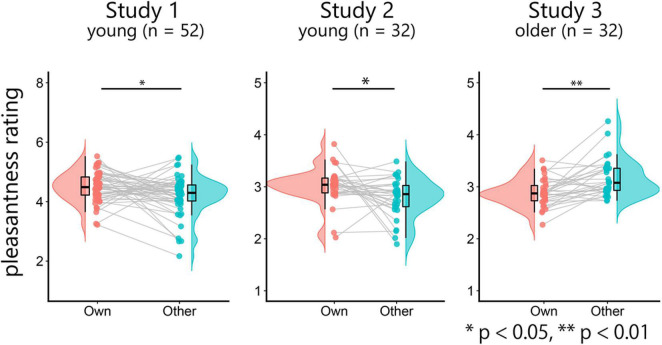
Raincloud plots of the mean pleasantness-rating scores for own-age and other-age faces. Note that study 1 used a different scale (1–8) compared to the other two studies (1–5).

We first investigated whether the rating scores for own-age faces were higher than those for other-age faces using paired *t*-tests (Figure 1). In studies 1 and 2, the rating scores for own-age faces were significantly higher than those for other-age faces (study 1, *t*(51) = –2.35, *p* = 0.02, *d* = –0.33, 95% confidence interval [CI] [–0.52, –0.04]; study 2, *t*(31) = –2.37, *p* = 0.02, *d* = –0.42, 95% CI [–0.43, –0.03]). On the other hand, the rating scores for other-age faces were significantly higher than those for own-age faces in study 3 (*t*(31) = –4.11, *p* < 0.001, *d* = –0.73, 95% CI [–0.41, –0.14]). These results suggest that young faces are perceived as more pleasant than older face.

**FIGURE 2:**
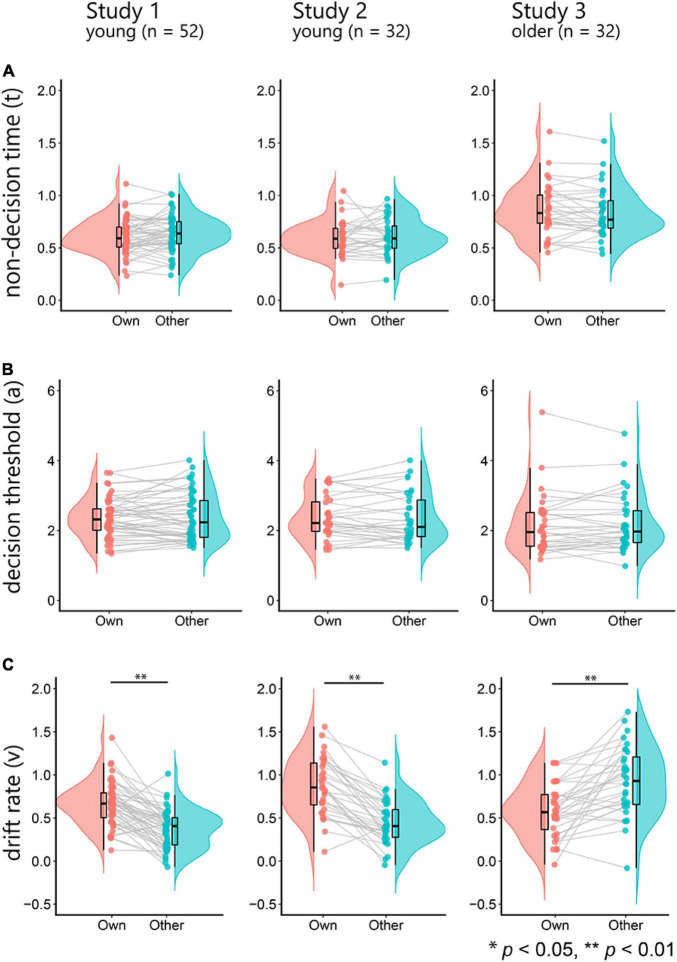
The top row depicts raincloud plots of the non-decision time (*t*) for own-age and other-age faces **(A)**. The left panel shows the results of study 1, the middle panel shows the results of study 2, and the right panel shows the results of study 3. The middle row depicts the decision threshold (*a*) for own-age and other-age faces **(B)**. The bottom row depicts the drift rate (*v*) for own-age and other-age faces **(C)**.

Next, for the results of the DDM, we investigated whether non-decision time (*t*), decision threshold (*a*), and drift rate (*v*) for own-age faces were significantly higher than those for other-age faces using paired *t*-tests (Figure 2). We found that the drift rates for own-age faces were significantly higher than those for other-age faces in studies 1 and 2 (study 1, *t*(51) = –8.38, *p* < 0.001, *d* = –1.16, 95% CI [–0.37, –0.23]; study 2, *t*(31) = –8.02, *p* < 0.001, *d* = –1.42, 95% CI [–0.53, –0.32]), but study 3 showed that the drift rates for other-age faces were significantly higher than those for own-age faces (*t*(31) = –4.76, *p* < 0.001, *d* = –0.84, 95% CI [–0.49, –0.20]) (Figure 2C). These results suggest that the drift rates for young faces were higher than those for older faces, regardless of the participants’ groups. We found no significant differences in the non-decision time and decision threshold (Figures 2A,B, all *p*-Values > 0.1). The ranges of the *-R* value in all parameter estimates indicated satisfactory convergence (study 1: 0.999–1.007, study 2: 0.999–1.007, and study 3: 0.999–1.019).

### Imaging Data

**FIGURE 3:**
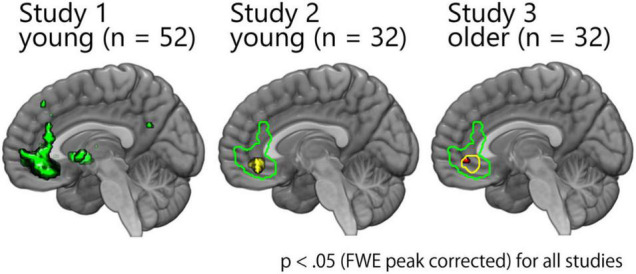
Activity of the overlapping cluster within the vmPFC was significantly correlated with the pleasantness-rating scores across the three studies. The results of studies 1 and 2 have been reported previously (Ito et al., 2016; Murakami et al., 2018) and are presented here only for display purposes. Consistent with these results, there was a significant correlation in the vmPFC among older participants.

First, we performed a parametric modulation analysis (Figure 3). The results of studies 1 and 2 have been reported previously (Ito et al., 2016; Murakami et al., 2018) and have been displayed here only for display purposes. Consistent with the results of studies 1 and 2, the results of study 3 showed a significant positive correlation between the pleasantness-rating score and the activity in the vmPFC (*x* = –2, *y* = 44, *z* = –6, *z*-value = 5.02, *k* = 9; *x* = –8, *y* = 30, *z* = –10, *z*-value = 4.75, *k* = 1) (Figure 3). Direct comparison between studies 2 and 3 showed no significant difference, indicating no age-related decline with regard to the representation of the subjective pleasantness of faces (Supplementary Results).

**FIGURE 4:**
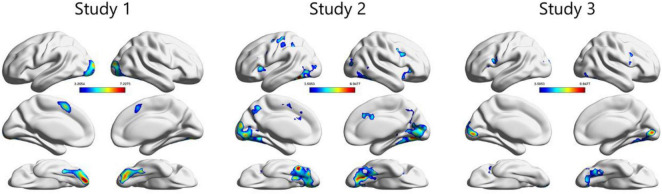
Across the three studies, the psycho-physiological interaction (PPI) analysis revealed that structures in the ventral visual areas, including those in the fusiform and occipital areas, demonstrated greater functional connectivity with the vmPFC for preferred faces compared with non-preferred faces. Here, the threshold of significance was set at *p* < 0.05 at the cluster level (FWE corrected for multiple comparisons) for display purposes (see Supplementary Table 2 for more information). The original statistical threshold was set at *p* < 0.05 at the peak level (FWE corrected for multiple comparisons) and the results are shown in Table 1.

In the PPI analysis, several brain regions, including structures within the ventral visual area, such as the fusiform and occipital gyri, showed greater functional connectivity with the vmPFC (Figure 4, Table 1, and Supplementary Table 2). Across the three studies, we identified clusters that overlapped with the FFA and OFA, as shown in previous study by Julian et al. (2012) and Neurosynth (Supplementary Figure 1). Direct comparison between studies 2 and 3 showed no significant difference, suggesting little evidence of age-related change of functional coupling between the vmPFC and ventral visual area with respect to value representation (Supplementary Results).

**TABLE 1:** Brain regions showing greater functional connectivity (preferred vs. non-preferred faces).

Region	Coordinates			*Z-*value	Cluster size
	*x*	*y*	*z*		
***Study 1***					
Left inferior occipital gyrus	–22	–86	–8	5.93	564
Right inferior occipital gyrus	18	–88	–4	5.64	415
Left fusiform gyrus	–36	–58	–14	4.96	33
Right fusiform gyrus	38	–48	–16	5.27	38
left SMA	–6	12	48	4.88	29
Left middle frontal gyrus	–44	2	30	4.58	5
***Study 2***					
Bilateral occipital gyrus	0	–96	6	5.27	8
Right fusiform gyrus	26	–74	–8	5.26	31
Left fusiform gyrus	–42	–58	–14	5.23	30
Right orbitofrontal cortex	28	30	–12	4.85	1
Left fusiform gyrus	–34	–80	–12	4.83	1
***Study 3***					
Right occipital gyrus	10	–86	2	5.00	23
Left occipital gyrus	–8	–88	–2	4.81	3
Left occipital gyrus	–14	–98	18	4.81	2

*The threshold of significance was set at p < 0.05 at the peak level (FWE corrected).*

We then performed a subtraction analysis to identify areas that showed greater activity for either own-age or other-age faces (Table 2 and Supplementary Table 3). Both young and older participants showed greater activation for own-age faces. For example, young participants showed greater activity in reward-related regions such as the vmPFC and ventral striatum, and older participants showed greater activity in the superior occipital gyrus.

**TABLE 2:** Brain regions showing significant activation for own-age faces and other-age faces.

Region (Brodmann’s Area)	Coordinates			*Z*-value	Cluster size
	*x*	*y*	*z*		
***Study 1***					
**Own-age face vs. other-age face**					
Left occipital gyrus	–26	–92	0	5.99	305
Left orbitofrontal cortex (extending to operculum)	–28	26	–14	5.67	49
Left brain stem (extending to the right side)	–4	–26	–10	5.57	71
Left thalamus (extending to right hemisphere)	–2	–18	8	5.44	101
Left inferior frontal gyrus	–40	18	22	5.18	40
Left fusiform gyrus	–44	–60	–12	5.04	59
Left anterior insula	–32	18	0	4.9	9
Left ventral striatum	–6	2	–6	4.77	2
Left inferior occipital gyrus	–42	–68	–4	4.64	1
Right fusiform gyrus	44	–52	–14	6.8	828
Right superior occipital gyrus	30	–74	28	5.38	110
Right orbitofrontal cortex (extending to operculum)	36	28	–2	5.11	99
Right middle cingulate gyrus	4	6	28	4.86	13
Right fusiform gyrus	48	–38	–12	4.83	1
Right ventral striatum	12	4	0	4.81	4
Right fusiform gyrus	42	–36	–14	4.78	2
Right cerebellum	8	–80	–28	4.63	1
**Other-age face vs. own-age face**					
no suprathreshold activation					
***Study 2***					
**Own-age face vs. other-age face**					
Left superior frontal gyrus	–16	40	50	4.79	5
Right orbitofrontal cortex	32	28	–16	5.13	6
Right orbitofrontal cortex	38	32	–12	4.7	2
**Other-age face vs. own-age face**					
no suprathreshold activation					
***Study 3***					
**Own-age face vs. other-age face**					
Right cuneus	12	–90	14	5.39	39
Right calcarine sulcus	14	–70	18	5.04	7
Right superior occipital gyrus	20	–92	22	4.85	6
**Other-age face vs. own-age face**					
no suprathreshold activation					

*The threshold of significance was set at p < 0.05 at the peak level (FWE corrected).*

### Hierarchical Drift-Diffusion Model Regression

Since the drift rate for young faces was significantly higher than that for older faces among the three studies, we applied an HDDMRegressor analysis to examine whether the trial-by-trial activity of the ROI could explain the drift rate for each dataset. For each ROI, the difference in percent signal change between chosen and unchosen faces (percent signal change for the chosen face – percentage signal change for the unchosen faces) in each trial was entered as the explanatory variable. A significant slope value for the explanatory variable implied that the signal of the ROI could explain the drift rate. The results are summarized in Table 3. Across the three studies, the slope value of the vmPFC was significantly positive for own-age faces. In addition, the results in study 1 showed a significantly positive slope value for the occipital gyrus among other-age faces. In study 2, the results also showed a significantly positive slope value for the fusiform and occipital gyrus among own-age faces and that for the vmPFC among other-age faces. In study 3, the results also showed a significantly positive slope value for the fusiform gyrus among other-age faces. The ranges of *-R* values for all parameter estimates indicated satisfactory convergence (study 1: 0.9996–1.0027, study 2: 0.9997–1.0004, and study 3: 0.9997–1.0006). To formally assess whether the vmPFC showed own-age bias (i.e., a greater slope for own-age faces relative to other-age faces), we performed a follow-up HDDMRegressor analysis that modeled an interaction term of vmPFC activity × face age. As expected, the regression coefficients for the interaction term were consistently positive across all three studies (study 1, β = 0.0129, *p* = 0.0665, 95% CI [–0.004, 0.029]; study 2, β = 0.006, *p* = 0.290, 95% CI [–0.016, 0.029]; study 3, β = 0.029, *p* = 0.023, 95% CI [0.0004, 0.057]). These results suggested trends toward the own-age bias in the trial-by-trial effect of vmPFC activity on the drift rate. A *post hoc* aggregation of results across the three studies indicated that the pooled mean of the regression coefficient was positive (mean = 0.0158, 95% CI [0.006, 0.026], between-study variance τ^2^ = 7.81 × 10^–5^). These results suggested that the vmPFC has greater role in the preferential choice of own-age faces. It should be noted that we did not find significant effects in other regions (left OFA, mean = 0.0017, 95% CI [–0.007, 0.01], between-study variance τ^2^ = 5.36 × 10^–5^; right OFA, mean = –0.003, 95% CI [–0.0224, 0.016], between-study variance τ^2^ = 2.88 × 10^–4^; left FFA, mean = –0.0038, 95% CI [–0.02, 0.013], between-study variance τ^2^ = 2.14 × 10^–4^; right FFA, mean = 0.001, 95% CI [–0.017, 0.019], between-study variance τ^2^ = 2.55 × 10^–4^). The supplementary analysis combining data from studies 2 and 3 also supports this notion (see Supplementary Results for details).

**TABLE 3:** Results of the hierarchical drift-diffusion model analysis.

Region	Own-age faces				Other-age faces			
	*slope*	*p-Value*	*credible interval*	*DIC*	*slope*	*p-Value*	*credible interval*	*DIC*
***Study 1***								
vmPFC	0.028	0.02*	0.003 0.052	7531.28	0.000	0.49	–0.024 0.024	8174.66
L fusiform	0.007	0.29	–0.018 0.032	7535.64	0.013	0.16	–0.014 0.036	8174.39
R fusiform	0.013	0.15	–0.012 0.04	7534.04	0.009	0.24	–0.016 0.034	8176.85
L occipital	0.004	0.38	–0.022 0.029	7536.52	0.011	0.19	–0.012 0.039	8176.17
R occipital	–0.004	0.39	–0.029 0.022	7535.30	0.025	0.02*	0.001 0.051	8173.16
***Study 2***								
vmPFC	0.051	0.001**	0.019 0.086	3824.80	0.037	0.01*	0.006 0.068	4436.40
L fusiform	0.027	0.05	–0.007 0.06	3830.81	0.000	0.50	–0.032 0.033	4442.82
R fusiform	0.034	0.03*	0.001 0.069	3829.01	–0.005	0.40	–0.038 0.029	4441.92
L occipital	0.023	0.09	–0.011 0.057	3831.20	0.005	0.39	–0.028 0.037	4440.96
R occipital	0.040	0.01*	0.006 0.076	3826.66	0.011	0.26	–0.021 0.042	4440.60
***Study 3***								
vmPFC	0.055	0.004**	0.014 0.095	2876.76	–0.007	0.37	–0.049 0.036	2585.06
L fusiform	–0.004	0.42	–0.046 0.037	2881.78	0.028	0.09	–0.012 0.069	2580.97
R fusiform	–0.013	0.25	–0.049 0.024	2883.79	0.021	0.17	–0.021 0.064	2583.41
L occipital	0.012	0.27	–0.026 0.049	2881.22	0.016	0.22	–0.024 0.058	2585.03
R occipital	0.022	0.12	–0.016 0.058	2880.26	0.035	0.04*	0.006 0.077	2582.20

*p < 0.05 and **p < 0.01; L, left; R, right.

## Discussion

Regardless of the age of the participants, the behavioral results demonstrated higher rating scores and higher drift rates for young faces than for older faces. These behavioral results showed no evidence of own-age bias in face preference, suggesting the important role of youth in face preference. Although neuroimaging results from the three datasets suggest a possibility that the vmPFC was associated with evidence of accumulation of own-age faces, results from study 1 did not reach significance and no robust evidence was provided. Importantly, we found no age-related decline in vmPFC responsiveness to subjective pleasantness of faces. The results of the present study suggest that preferential choice of face is less susceptible to own-age bias across the life span of individuals.

Across the three studies, the behavioral results consistently showed a higher drift rate for young faces, suggesting the role of youth rather than own-age bias. The supplemental analysis showed that the difference in rating scores of the young face pairs, as calculated by subtracting the score for face B from that for face A, was significantly larger than that of the older face pairs (Supplementary Figure 2). Thus, the larger difference in the subjective value between items in the young pairs which is originated from the nature that the young face is more pleasant than older face was related to this young-age effect. Another possibility is that the difference of salience in young face pairs was larger than that of the older face pairs, which might make it easier to make preferential choices among young face pairs. Consistent with this idea, differences in salience between young and older faces have been implicated in previous psychological studies (Ebner, 2008), and this idea is in line with the previously established evidence that salience benefits value-based choice (Towal et al., 2013; Chen et al., 2021). It is plausible that features closely linked to young faces, such as facial fullness and smooth and clear skin, are associated with these behavioral patterns (Buss and Barnes, 1986; Coleman and Grover, 2006; Popenko et al., 2017; Sakano et al., 2021).

Although the current findings are inconclusive and future studies are required to corroborate them, the overall pattern of the HDDM regression analysis suggests that the vmPFC exhibits an own-age bias in evidence accumulation. Since the ROI of the vmPFC was obtained from a previous study that showed that the vmPFC played a role in preference-related value representation (Lebreton et al., 2009), a straightforward interpretation is that the preferential choice of own-age faces mainly relies on the comparison of the subjective values, whereas the preferential choice of other-age faces also relies on other factors. This view is supported by previous literature that argued that various factors such as familiarity, preference, salience, or more available prototypes may underlie the differences in the processing of in-group and out-group faces (Ebner et al., 2011b, 2013).

Value-based choice consists of two stages: valuation and selection (Rangel et al., 2008; Kable and Glimcher, 2009). Previous research focusing on the neural substrates of the two-stage processes demonstrated that the initial valuation process is supported by the vmPFC, and that the subsequent selection processes (i.e., integration and read-out) are supported by the dorsolateral prefrontal cortex and posterior parietal cortex, respectively (Domenech et al., 2017). Thus, it is also possible that the perceptual expertise of own-age faces enables the vmPFC to work solely on the valuation process, whereas participants showed less perceptual expertise for other-age faces, which required other regions such as the fusiform and occipital gyri to support the vmPFC. It should be noted that the vmPFC activity applied in the HDDM regression analysis was assessed during the pleasantness-rating task but not during the choice task. Thus, the differential contributions of the vmPFC may be due to the different processes in the valuation stage. Future studies assessing vmPFC activity during both the rating task and choice task are warranted to formally investigate the role of the vmPFC in valuation and subsequent selection processes.

Importantly, regardless of the age of the participants, we found that the vmPFC parametrically represented the subjective value of faces, and showed stronger functional connectivity with visual areas when participants were presented with faces that they preferred. It should be noted that direct group comparisons between the young participants (study 2) and older participants (study 3) showed no significant difference (Supplementary Results). These results suggest that the function of computing subjective values of faces can be maintained in older participants, and that the own-age effect identified in the vmPFC was not attributable to age-related functional decline. Recent reports employing a mixed-lottery choice task that required participants to calculate the expected value of the options have indicated the influence of aging on value-based decision making (Su et al., 2018; Chen et al., 2021), which is considered to be followed by a decline in dopaminergic modulation and fronto-striatal network functioning (Samanez-Larkin and Knutson, 2015). Compared to such processes, the preference-related value representation of faces appears to be less susceptible to aging.

The functional network of the vmPFC and ventral visual stream comprises a part of an extended face processing system in the human brain (Haxby et al., 2000; Elbich et al., 2019). Previous studies have revealed the contribution of the ventral visual stream to facial attractiveness (Kranz and Ishai, 2006; Winston et al., 2007; Chatterjee et al., 2009; Pegors et al., 2015). More directly, another fMRI study demonstrated a functional coupling between the vmPFC and fusiform gyrus in representing the subjective value of a T-shirt (Lim et al., 2013). Taken together with the function of the vmPFC in modality- and category-independent value representation (Chib et al., 2009; Lebreton et al., 2009; Ishizu and Zeki, 2011; McNamee et al., 2013), the representation of the subjective value of a face may be supported by two valuation systems: the BVS which represents subjective value in a domain-general manner subserved by the vmPFC (Lebreton et al., 2009) and the extended BVS which represents subjective value as a domain-specific manner subserved by the functional coupling between the BVS and sensory area.

The function of the BVS is to reflect both the value, that is explicitly revealed by participants (e.g., rating), and preference, which is revealed in binary choices. The BVS often includes reward-related regions, such as the vmPFC and ventral striatum (Lebreton et al., 2009; Ito et al., 2016; Murakami et al., 2018). On the other hand, the role of the extended BVS is to serve as a functional network between the BVS and sensory areas, which are involved in the representation of subjective values. Thus, we argue that the functional network among the vmPFC, fusiform gyrus, and occipital gyrus identified in this study meets this requirement and can be considered as the extended BVS for face preference. Importantly, based on recent evidence showing functional coupling of the vmPFC and the fusiform gyrus in value computation of brand names (Zhang et al., 2021), the role of the extended BVS might not be limited to the face. In fact, a previous study that employed music as a stimulus identified an extended BVS for auditory preference (Salimpoor et al., 2013). The researchers found a functional coupling between the ventral striatum and the auditory cortex, which represented subjective values of musical excerpts. A recent understanding of how subjective value was formed through bottom-up processing (Iigaya et al., 2020; Pham et al., 2021) indicates that the extended BVS might serve as a gateway for domain-specific value computation or attribute value computation (Lim et al., 2013).

A natural interpretation of the higher pleasantness-rating scores for young faces found in both age groups is that young faces are more rewarding than older faces, regardless of participants’ age. Previous research which proposed the three-dimensional model of social inference from faces showed that the ‘youthful-attractiveness’ factor consistently emerged from an unconstrained set of face stimuli (Sutherland et al., 2013). The role of youthfulness in impression formation and social interaction has long been advocated in previous seminal reviews (Buss and Schmitt, 1993; Rhodes, 2006). Supporting these findings, evidence from the field of esthetic surgery revealed age-related facial structural changes, such as progressive bone resorption, decreased tissue elasticity, and loss of facial fullness (Coleman and Grover, 2006), and indicated that the fullness of the face is directly linked to attractiveness (Popenko et al., 2017). Facial pleasantness appears to be independent of perceptual expertise or familiarity with one’s own age group.

The results of the whole-brain analysis showed that own-age faces elicited stronger activation than other-age faces, but not vice versa. One possibility is that the stronger activation for own-age faces might reflect a differential gaze pattern to own-age and other-age faces. This view is supported by a previous eye-tracking study that revealed that people see own-age faces longer than other-age faces (Ebner et al., 2011c; He et al., 2011). Perhaps these differential gaze patterns might reflect perceptual expertise or familiarity with own-age faces acquired through recent experiences with one’s own age group (Chiroro and Valentine, 1995; Anastasi and Rhodes, 2005; Rhodes and Anastasi, 2012). However, although we found greater activity of the visual area for own-age faces in studies 1 and 3, this finding was not replicated in study 2 and further investigations are required in this regard. Furthermore, we did not collect eye-tracking data in the present study. Future studies that combine neuroimaging techniques with eye tracking could provide direct evidence that disentangles causes related to own-age-specific brain activity. Alternatively, subjective similarity to one’s own-age faces might be related to differential activity. Ebner et al. (2011a) found the contribution of the vmPFC to own-age face processing (Ebner et al., 2011a), supporting the previous evidence that the vmPFC shows higher activation when thinking about similar than dissimilar others (Amodio and Frith, 2006; Mitchell et al., 2006; Van Overwalle, 2009). Given its contribution to various aspects of social cognition, such as emotional empathy, social reputation, moral judgment, and value-based decision making (Saxe, 2006; Forbes and Grafman, 2010; Ito et al., 2011, 2015; Delgado et al., 2016; Kawasaki et al., 2016; Hiser and Koenigs, 2018; Yoon et al., 2018; Suzuki and O’Doherty, 2020; Pessiglione and Daunizeau, 2021) as well as its multifaceted anatomical connections (Rolls and Grabenhorst, 2008; Grabenhorst and Rolls, 2011; Riga et al., 2014), dissociable neural populations would contribute to own-age face recognition and facial preference.

## Data Availability Statement

The data supporting the conclusions of this article will be made available by the authors, without undue reservation.

## Ethics Statement

The studies involving human participants were reviewed and approved by the Ethical Committee of Hokkaido University and the Ethical Committee of Tohoku University. The patients/participants provided their written informed consent to participate in this study.

## Author Contributions

AI, KY, and RA contributed to conception, design of the study, and performed the statistical analysis. AI, TF, IK, AH, AU, SS, SM, and ST collected the data. AI organized the database. AI wrote the first draft of the manuscript. KY and RA wrote sections of the manuscript. EM supervised the study. All authors contributed to manuscript revision, read, and approved the submitted version.

## Conflict of Interest

The authors declare that the research was conducted in the absence of any commercial or financial relationships that could be construed as a potential conflict of interest.

## Publisher’s Note

All claims expressed in this article are solely those of the authors and do not necessarily represent those of their affiliated organizations, or those of the publisher, the editors and the reviewers. Any product that may be evaluated in this article, or claim that may be made by its manufacturer, is not guaranteed or endorsed by the publisher.

## Footnotes

1
https://www.python.org/

2
http://marsbar.sourceforge.net/

## Supplementary Material

Supplementary Figure 1 Overlapping areas (shown in pink) between significant clusters identified in the PPI analysis of each study (studies 1–3, from the top to the bottom, shown in red), clusters of the FFA and OFA reported by Julian et al. (2012) (left panels), and clusters of the FFA and OFA identified in the “ffa” *Z*-maps obtained from Neurosynth (right panels) (shown in blue). Study 1 **(a,b)** and study 2 **(c,d)** revealed an overlap in both hemispheres, and study 3 **(e,f)** showed overlap in the right hemisphere. Here, the threshold of significance was set at *p* < 0.05 (cluster-level FWE corrected).

Supplementary Figure 2 Differences in rating scores among own-age face pairs, as calculated by subtracting scores for face B from those of face A and those of the other-age face pairs.

Supplementary Figure 3 From top to bottom: study 1 **(a)**, study 2 **(b)**, and study 3 **(c)**. From left to right: non-decision time (*t*), decision threshold **(a)**, and drift rate (v). In study 1, parameters were calculated based on stimulus gender (female and male) and stimulus age (own and other). In studies 2 and 3, parameters were calculated based on participant gender (F and M), stimulus gender (female and male), and stimulus age (own and other). F, female participants; M, male participants.
