# Supplementary figures and images for: The Role of the Ventromedial Prefrontal Cortex in Preferential Decisions for Own- and Other-Age Faces
Source: Front Psychol. 2022 Mar 11;13:822234. doi: 10.3389/fpsyg.2022.822234 (PMC8962742; doi:10.3389/fpsyg.2022.822234)

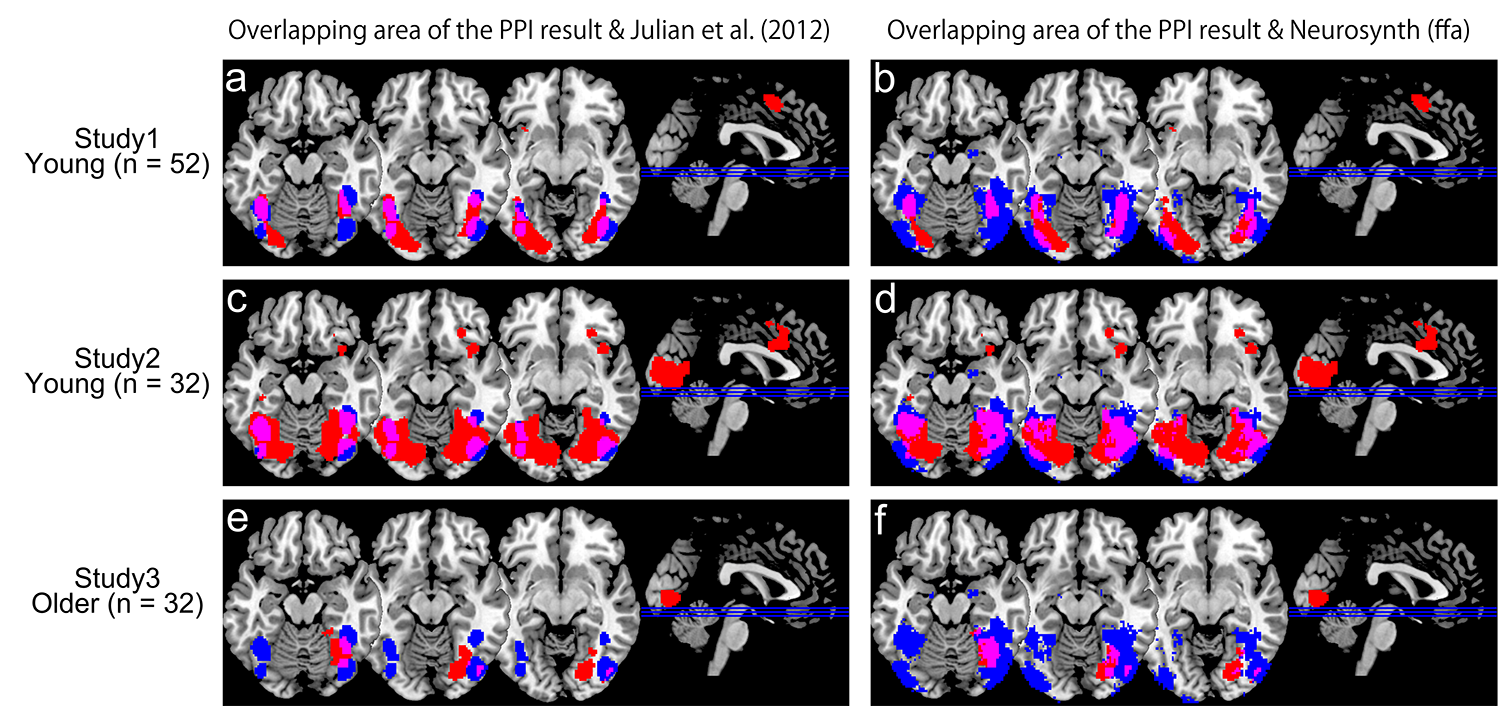

Supplement: Supplementary Figure 1 — Overlapping areas (shown in pink) between significant clusters identified in the PPI analysis of each study (studies 1–3, from the top to the bottom, shown in red), clusters of the FFA and OFA reported by Julian et al. (2012) (left panels), and clusters of the FFA and OFA identified in the “ffa” Z-maps obtained from Neurosynth (right panels) (shown in blue). Study 1 (a,b) and study 2 (c,d) revealed an overlap in both hemispheres, and study 3 (e,f) showed overlap in the right hemisphere. Here, the threshold of significance was set at p < 0.05 (cluster-level FWE corrected). [file Image_1.tif]

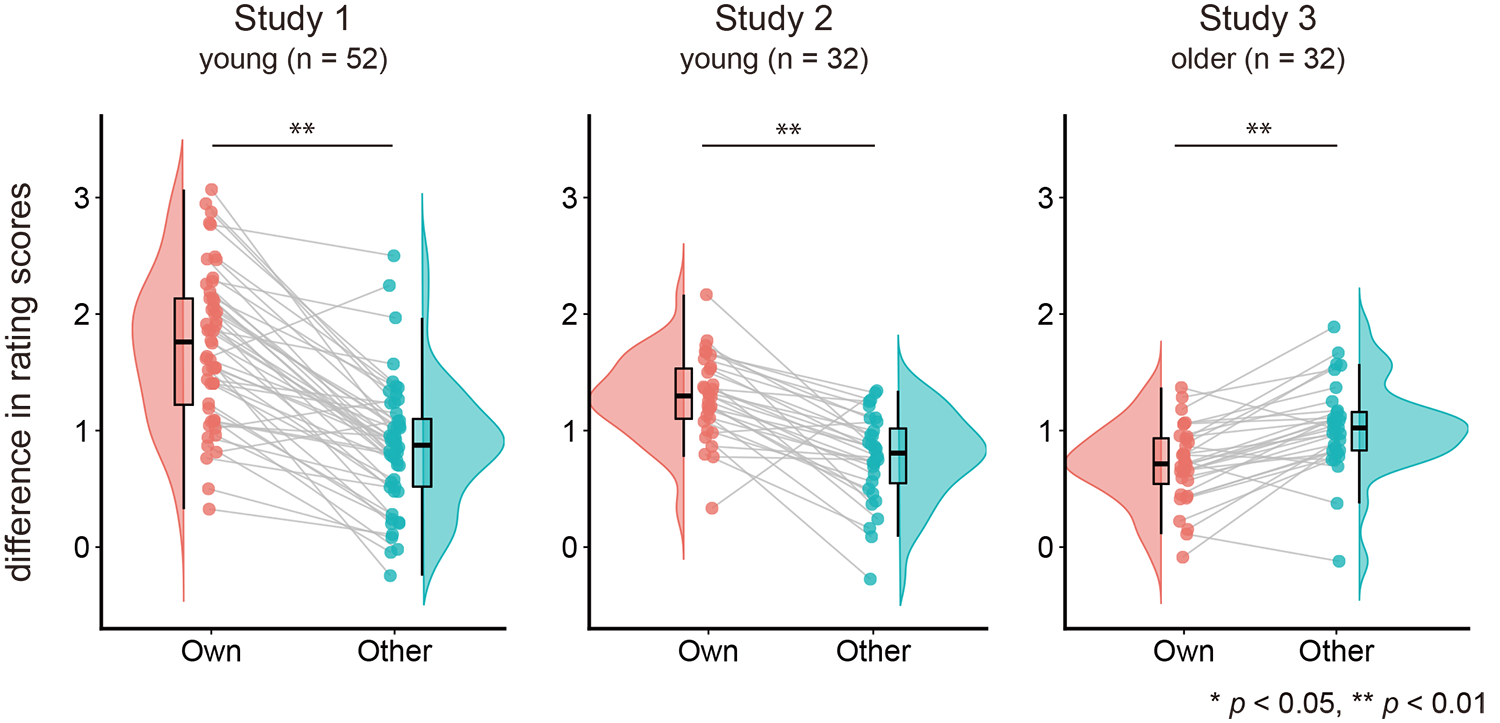

Supplement: Supplementary Figure 2 — Differences in rating scores among own-age face pairs, as calculated by subtracting scores for face B from those of face A and those of the other-age face pairs. [file Image_2.tif]

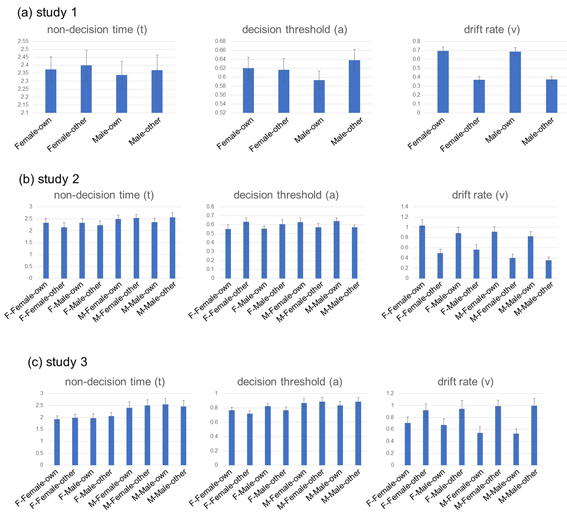

Supplement: Supplementary Figure 3 — From top to bottom: study 1 (a), study 2 (b), and study 3 (c). From left to right: non-decision time (t), decision threshold (a), and drift rate (v). In study 1, parameters were calculated based on stimulus gender (female and male) and stimulus age (own and other). In studies 2 and 3, parameters were calculated based on participant gender (F and M), stimulus gender (female and male), and stimulus age (own and other). F, female participants; M, male participants. [file Image_3.jpg]
