# Supplementary material for: The Role of the Ventromedial Prefrontal Cortex in Preferential Decisions for Own- and Other-Age Faces
Source: Front Psychol. 2022 Mar 11;13:822234. doi: 10.3389/fpsyg.2022.822234 (PMC8962742; doi:10.3389/fpsyg.2022.822234)
Supplement: Supplementary file 4 [file Data_Sheet_1.pdf]

Supplementary Results

Direct comparison of behavioral data between studies 2 and 3

Since studies 2 and 3 were performed under the same environment (i.e., the same task, rating buttons, and MRI), we performed supplementary analyses to directly compare the behavioral and neuroimaging data.

Pleasantness rating score

The pleasantness rating scores of the participants from studies 2 (young participants) and 3 (older participants) were analyzed using a two-way repeated-measures ANOVA with the age of stimulus face (own-age and other-age) as the within-participant factor and the age of the participants (young and older) as the between-participant factor. The results showed significant interaction ($F[1,62] = 18.31$, $p < .001$), but showed no significant main effect of the age of stimulus face ($F[1,62] = .13$, $p = .72$) and the age of the participants ($F[1,62] = 3.9$, $p = .052$).

Reaction time in the pleasantness rating task

The reaction times during the pleasantness rating task in studies 2 (young participants) and 3 (older participants) were analyzed using a two-way repeated-measures ANOVA with the age of stimulus face (own-age and other-age) as the within-participant factor and the age of the participants (young and older) as the between-participant factor. This ANOVA showed significant main effect of the age of stimulus face ($F[1,62] = 4.19$, $p < .05$), but showed no significant main effect of the age of the participants ($F[1,62] = 1.05$, $p = .31$) and interaction ($F[1,62] = .35$, $p = .56$).

Reaction time in the preference choice task

The reaction time during the preference choice task from studies 2 (young participants) and 3 (older participants) were analyzed using a two-way repeated-measures ANOVA

with the age of stimulus face (own-age and other-age) as the within-participant factor and the age of the participants (young and older) as the between-participant factor. This ANOVA showed significant interaction ($F[1,62] = 16.64$, $p < .001$), but showed no significant main effect of the age of stimulus face ($F[1,62] = .17$, $p = .68$) and the age of the participants ($F[1,62] = .09$, $p = .77$).

Direct comparison of neuroimaging data between studies 2 and 3

Parametric modulation analysis

We performed a two-sample t-test to isolate regions which showed significant difference of activity between the young and the older. For this analysis, the threshold of significance was set at $p < 0.05$ (family-wise error corrected for multiple comparisons at the cluster level). The results showed no suprathreshold activation, suggesting that the function of representing the subjective value of faces is retained through aging.

PPI analysis

We performed a two-sample t-test to isolate regions which showed significant differences in functional connectivity between the young and the older. For this analysis, the threshold of significance was set at $p < 0.05$ (family-wise error corrected for multiple comparisons at the cluster level). The results showed no suprathreshold activation and suggest that there is no obvious age difference in the functional coupling between the vmPFC and visual area.

The effect of age and gender on HDDM parameters

Although we did not have any a priori hypothesis concerning the effect of the participants' gender and stimulus gender, as an exploratory post-hoc analysis, we decomposed the behavioral data based on the participants' gender and stimulus gender and performed ANOVAs. Since study 1 only included male participants, we performed a two-way repeated-measures ANOVA with the age of stimulus face (own-age and other-age) and

gender of stimulus face (female and male) as within-participant factors for non-decision time (a), decision threshold (t), and drift rate (v), respectively (Supplementary Fig. 3a). The results showed that ANOVAs for the non-decision time and decision threshold showed no significant main effects or interactions (all *P*s > .1). On the other hand, ANOVA for the drift rate showed significant main effect of the age of stimulus face ($F[1,51] = 78.69$, $p < .001$), but showed no significant main effect of the gender of stimulus face ($F[1,51] = .002$, $p = .96$) and interaction ($F[1,51] = .03$, $p = .86$). Consistent with the original analysis using t-tests, these results showed that evidence accumulation is faster for young faces compared to that for older faces.

As studies 2 and 3 were performed in the same environment and both studies included female and male participants, the data from these studies were concatenated and analyzed by a four-way repeated-measures ANOVA with the age of stimulus face (own-age and other-age) and the gender of stimulus face (female and male) as within-participant factors, and with participant age (young and older) and participant gender (female and male) as between-participant factors. The ANOVA was performed for non-decision time (a), decision threshold (t), and drift rate (v), respectively (Supplementary Fig. 3b and 3c). The ANOVA for non-decision time showed a significant three-way interaction (stimulus age*participant age*participant gender, $F[1,60] = 5.09$, $p = .028$) and significant main effect of participant gender ($F[1,60] = 4.03$, $p = .049$). This ANOVA showed no other main effects or interactions (all *P*s > .1). The ANOVA for decision threshold showed a significant three-way interaction (stimulus age*participant age*participant gender, $F[1,60] = 17.99$, $p < .001$) and significant main effect of participant age ($F[1,60] = 31.67$, $p < .001$). This ANOVA showed no other main effects or interactions (all *P*s > .05). Although the original analysis using t-tests did not show the effect of stimulus age, these results suggest that non-decision time and decision threshold can be affected by the interaction between the attributes of the perceiver (i.e., participant's age and gender) and face age. The ANOVA for the drift rate showed a significant two-way interaction (stimulus age*participant age, $F[1,60] = 71.34$, $p < .001$) but showed no other main effects or interactions (all *P*s > .1). Consistent with the original analysis using t-tests, this supports the existence of the young-age effect.

Additional HDDMRegressor analysis examining the effects of age and gender

Since the original HDDMRegressor analyses (see the main manuscript for details) was performed to examine the neural correlates of the own-age bias, only the face age (i.e., own-age face and other-age face) and region of interest (ROI) were considered. Here, we performed additional HDDMRegressor analyses to examine the effects of age, gender, and ROI. As study 1 employed only young male participants, the stimulus age, stimulus gender, and ROI were included as explanatory variables as follows: drift rate ~ ROI*stimulus age*stimulus gender. In this model, we included all possible interactions and main effects. This analysis was performed for each ROI (vmPFC, left and right FFA, left and right OFA).

As studies 2 and 3 were performed in the same environment (i.e., the same rating buttons and MRI), the data from these studies were concatenated for the analysis. Then, we constructed a comprehensive model that included the effects of age and gender of stimulus and participants (i.e., 2×2 factors) and performed HDDMRegressor analysis using the following model: drift rate ~ ROI*stimulus age*stimulus gender*participant age*participant gender. Here, all possible interactions and main effects were included, and the analysis was performed for each ROI (vmPFC, left and right FFA, left and right OFA). For those regressions, three chains were run, each containing 2,000 samples, and the first 1000 samples in each run were discarded to improve convergence. The slope in each effect for estimating the DDM parameters were calculated for each simulation, and the average values and significance were reported based on 3,000 samples. All the other methodologies were the same as those in the original HDDMRegressor analysis reported in the main manuscript.

Significant effects ($p < .05$) are reported in Supplementary Table 4. In study 1, we found the effect of stimulus age which indicated a higher drift rate when young age faces were presented. The interaction of vmPFC activity and stimulus age was marginally significant (slope = .013, 95% CI [-0.004 0.031], p = .059). In studies 2 and 3, consistent with the original analysis, we found a significant interaction between vmPFC activity and stimulus age (slope = .019, 95% CI [0.003 0.038], p = .02), and this interaction was not found in other regions (all Ps > .05). The interaction of the stimulus age and participant

age was found in every ROI ($p < .001$), suggesting that the drift rate for the young face is higher than that for the older face. We also found significant effects of stimulus gender and stimulus age across the ROIs, suggesting that the drift rates for own-age and female faces are higher than those for other-age and male faces. The ranges of $\hat{R}$ values for all parameter estimates indicated satisfactory convergence (study 1: 0.9995–1.0083; studies 2 and 3: 0.9995–1.0166).

Mixed-effects analysis using concordance score

We performed an additional analysis that quantifies the concordance of performance between the two tasks (i.e., ratings in the pleasantness rating task and choices in the preference choice task). First, for each face pair presented in the preference choice task, we assigned arbitrary labels (A and B) to distinguish the identities of the two faces. Second, we coded a participant's trial-wise binary choice in the preference choice task as 1 if the participant chose face A and 0 if chose face B. Third, we computed a rating difference of each face pair as "pleasantness rating score for face A – pleasantness rating score for face B," on the basis of the participant's behavior in the pleasantness rating task. We used the raw signed score as the rating difference (e.g., if pleasantness rating score for face A was 4 and that for face B was 5, the difference was –1). Lastly, we fit a mixed-effects model, with the participants' trial-wise choices as the dependent variable and the rating differences as the primary independent variable. The model also included interaction terms (rating difference-by-the age of stimulus face and rating difference-by-the gender of stimulus face), random intercept effects of participants and stimuli, and a random slope effect of participants on the rating difference. Namely, the model equation was:

Trial-wise choice ~ 1 + rating difference + the age of stimulus face + the gender of stimulus face + rating difference : the age of stimulus face + rating difference : the gender of stimulus face + (1 + rating difference | participant ID) + (1| stimulus ID)

The coefficient for the rating difference term can be regarded as a concordance score, with a larger value indicating a greater concordance of behaviors between the two tasks. Furthermore, the coefficient for the rating difference-by-the age of stimulus face

interaction term reflects the difference of the degrees of concordance between own-age and other-age faces (i.e., a positive value indicates a greater concordance for own-age relative to other-age faces). We performed this mixed-effects analysis for each study. Thus, we built three models in total. Across the three studies, the effect of the rating difference was significantly positive, suggesting the concordance between the two tasks. Also, the results from study 1 and 2 showed young-age effect (Supplementary Table 5). However, the results from study 3 did not show the young-age effect. Although future studies are needed, these results may suggest age-related change of face evaluation system.
