# Supplementary material for: The Role of the Ventromedial Prefrontal Cortex in Preferential Decisions for Own- and Other-Age Faces
Source: Front Psychol. 2022 Mar 11;13:822234. doi: 10.3389/fpsyg.2022.822234 (PMC8962742; doi:10.3389/fpsyg.2022.822234)
Supplement: Supplementary file 5 [file Table_1.docx]

**Supplementary Table 1**

Reaction times (RTs) in the preference choice task.

| Study | N | Own age face | Other age face | t-value | 95% CI | p-value |
| --- | --- | --- | --- | --- | --- | --- |
| Study 1 | 52 | 1.81 ± 0.60 | 2.03 ± 0.78 | -3.37 | [-0.35 -0.09] | *p* < .01 |
| Study 2 | 32 | 1.73 ± 0.70 | 1.98 ± 0.85 | -3.08 | [-0.41 -0.08] | *p* < .01 |
| Study 3 | 32 | 2.03 ± 1.23 | 1.86 ± 0.90 | 2.68 | [0.05 0.36] | *p* = .012 |

CI: Confidence Interval
